# The Mediating Role of Organizational Identification on Sustainable Human Resources Management and Organizational Citizenship Behavior’s Relationship

**DOI:** 10.5812/ijpr-140447

**Published:** 2023-12-31

**Authors:** Shiva Sheikhi, Nazila Yousefi

**Affiliations:** 1 Department of Pharmacoeconomics and Pharma Management, School of Pharmacy, Shahid Beheshti University of Medical Sciences, Tehran, Iran

**Keywords:** Sustainable Human Resource Management, Organizational Identification, Organizational Citizenship Behavior, Pharmaceutical Industry

## Abstract

**Background:**

There is a general theme in studying employees in the research and development (R&D) department individual performance studies, where tremendous attention has been paid to innovation performance compared to behavioral and particularly extra-role behavior of employees in this department.

**Objectives:**

This study investigates the relationship between sustainable human resource management (s-HRM) and organizational citizenship behavior (OCB) through the mediating role of organizational identification (OI) in R&D employees.

**Methods:**

A standard questionnaire was used to evaluate s-HRM, OI, and OCB. Five hundred questionnaires were delivered to all employees of the research and development departments of 59 Iranian pharmaceutical companies, and finally, 316 completed questionnaires were collected.

**Results:**

The results of data analysis with WarpPls software revealed a positive and significant relationship between s-HRM and OI, as well as OI and OCB. Investigating the mediating role of OI showed that OI partially mediates the relationship between s-HRM and OCB. The model was checked in terms of its fit indices, which were evaluated as favorable.

**Conclusions:**

The findings suggest that s-HRM improves employees' willingness to go beyond their defined job description to display in OCB. Additionally, they imply that strengthening OI can improve OCB in employees.

## 1. Background

Nowadays, much attention has been devoted to the remarkable role of the pharmaceutical industry in the world economy, which provides essential healthcare products and services. This industry is important due to its innovative and research-intensive nature, as it continuously strives to develop new and effective drugs to improve health outcomes (1-3). These developments have resulted in evolving the approach towards human resource management from a strategic to a sustainable approach as well. In this sustainable approach, human resource management strategies are designed and carried out in a way that optimally meets the current needs of the firm and society without jeopardizing their ability to respond to future needs (4).

The recent years have witnessed remarkable growth in the implementation of sustainable human resource management (s-HRM) through the development of appropriate human resource policies and strategies (5). S-HRM is an approach used for managing human resources that focuses on creating sustainable competitive advantages through the development of a highly skilled, motivated, and committed workforce by emerging as a critical factor in the success of companies (6). S-HRM is particularly relevant in the pharmaceutical industry, where the success of R&D initiatives depends on the availability of highly skilled personnel with the necessary expertise and experience.

 In the context of the present study, the focus has shifted towards the development of generic drugs, which are more affordable and accessible to the general public. This shift in focus has necessitated a change in the R&D process, moving operations in the development process such as proving bioequivalence to the original drugs instead of conducting full-scale clinical trials (7).

According to social exchange theory, employees engage in OCB when they perceive that they have received something in return for their positive behaviors, such as recognition, support, and opportunities for development. In the context of s-HRM, the organization invests in the employees' well-being, development, and growth, which fosters a sense of reciprocity and obligation among employees to enhance behaviors that are beneficial to the organization beyond their job duties (8).

Nevertheless, OCB includes voluntary behaviors of employees that are not part of the official duties and are not directly considered as employees' duties by the official reward system and increase the overall effectiveness of the organization. Hence, organizational citizenship behavior (OCB) is considered here as voluntary extra-role behavior by an employee, which, despite its voluntary nature, benefits the effective functioning of the firm (9, 10). Organizations can benefit from using OCB to boost productivity, boost employee morale, encourage teamwork among coworkers, and generate a favorable work atmosphere, all of which can benefit both employees and companies (11).

In the case of s-HRM, the organization's investments in the employees' well-being, development, and growth foster a sense of reciprocity and obligation among employees to demonstrate behaviors beneficial to the organization beyond their formal duties, i.e., OCB (12). As the stability of the pharmaceutical industry depends on the research and development teams, it is essential to sustainably keep them, noting their work-life balance and health (13).

However, there is still much to uncover about the process through which sustainable HRM practices bring about performance outcomes at the individual level, especially if we consider HRM’s part to be the productive use of employees to achieve the objectives of the firm as well as the incentivizing them to display extra-role behavior. Building on previous work by Shen and Benson (2014), the present study aims to investigate whether or not and in what way sustainable human resource management practices lead to the participation of employees in organizational citizenship behavior that benefits the organization (14).

To the best of our knowledge, and despite the critical role of s-HRM, OI, and OCB in the success of pharmaceutical companies, there is limited research on the relationship between these factors. Therefore, this research aimed to examine the relationship between s-HRM, OI, and OCB, particularly in the context of pharmaceutical companies using “social exchange” and “organizational identification” theories. Based on the same theoretical framework, Wei et al. discussed that organizational identification (OI) has a positive relationship with employees' organizational citizenship behavior (15). On the other hand, organizational identification theory posits that employees' identification with the organization is influenced by their perception of shared values, goals, and identity with the organization (16). OI is generally referred to as the extent to which a person identifies with the same characteristics and values that characterize the organization to which they belong and is another critical factor that influences the success of companies (17); when it comes to s-HRM, the organization's commitment to sustainable and responsible HR practices can reinforce employees' identification with the OI, leading to a greater sense of connection and commitment to the objectives and principles of the organization as it creates a sense of belonging as well as commitment among employees, which, in turn, enhances their performance and productivity.

As previous studies have mainly focused on the direct relationship between s-HRM and OCB, without considering the mediating role of other variables, the contribution of the current study is examining the way that other variables mediate this relationship. Furthermore, the previous research has mainly focused on linear relationships, which does not always accurately demonstrate the real world (18). At the same time, the current work examines the non-linear or warped relationships that may exist between these variables. In addition, the lack of empirical research on the relationship between s-HRM and OCB is addressed in the context of developing countries is another contribution. Then, the findings provide important insights for pharmaceutical companies in Iran seeking to improve their R&D department's performance and competitiveness through effective HRM practices.

Overall, this study aims to advance our understanding of how s-HRM practices influence employee behavior, specifically OCB, by examining the mediating role of OI. The findings will offer valuable insights for both academia and pharmaceutical companies, guiding the development and implementation of effective HRM strategies to enhance employee performance, well-being, and organizational success.

### 1.1. Theory and Hypotheses Development

S-HRM practices are identified as human resource systems or practices that increase the organization's ability to maximize profits while reducing harm to employees (i.e., the social dimension of the triple bottom line) (19, 20). Employee harm of work refers to the reduction of psychological, social, and occupational health and welfare for various stakeholders (such as employees, their family members, and communities) (21).

### 1.2. Sustainable Human Resource Management and Organizational Identification

Based on previous research, s-HRM practices have been found to promote employee well-being and job satisfaction, which in turn lead to higher levels of OI. Therefore, it is expected that employees who perceive their organization as adopting s-HRM practices will have a stronger sense of identification with their organization (22, 23). As previously mentioned, both the theories of social identity and social exchange can provide insights into the explanation of how s-HRM relates to employees' OCB. OI has been used in social exchange and social identity-based studies to explain how HRM practices may induce a higher sense of OI (14, 24).

Social identity theory suggests that people's identification with the organization is stronger when the organization has a good reputation in their eyes because membership in that organization increases their self-esteem (25).

In other words, by implementing s-HRM, the organization can benefit its internal stakeholders and create a positive external reputation for itself. This positive reputation makes individuals proud to be employed in their organization and strengthens their OI (26).

Social exchange theory (27) has also been used to explain why HRM practices may prompt employees to develop stronger OI and, in turn, take the form of positive organizational behaviors. Building on the mentioned theories, we suggest that using s-HRM practices by organizations that pay attention to employees' needs and resolve their concerns leads employees to reciprocate in the format of OI. As an illustration, individuals working for an organization that follows employment, health, and safety regulations and promotes equal opportunity for them are more likely to commence psychologically investing in the organization and develop a higher level of OI. Therefore, we propose:

Hypothesis 1: Sustainable human resource management has a direct relationship with organizational identification.

### 1.3. Organizational Identification and Organizational Citizenship Behavior

Based on social identity theory, individuals who identify with their organization are more likely to display behaviors such as OCB. Therefore, it is expected that employees who have a strong sense of identification with their organization will engage in more organizational citizenship behavior.

OI includes two basic motivations: (A) the need for self-categorization (the degree to which the employee considers himself to belong to the organization) and (B) the need for self-enhancement (a sense of pride in belonging to the organization or a sense of approval in it). In terms of its relationship with OCB, OI has a long history of influencing organizational citizenship behavior (10). In particular, an employee who has a stronger identity connection with their organization may have a stronger motivation to think about and address workplace problems from the perspective of group interests. Such employees define themselves based on a collective identity orientation and prefer to be "good citizens" (28).

A good organizational citizen is an individual who exhibits individual, voluntary, and conscious behaviors that are not directly and openly identified by organizational reward systems and organizational performance evaluation systems but have a very impressive effect on organizational effectiveness overall (29).

For a long time, researchers have distinguished between in-role performance and extra-role performance through OCB. Extra-role performance refers to job behaviors beyond the official roles of employees, which are optional and are usually not considered a reward or payment in the organization's official reward system (30).

Since OI includes the basic definition of existence (individual and organizational identities), it has a unique value in explaining individual attitudes and behaviors in organizations. Due to the complexity of work in workplaces, it is often necessary for employees to go beyond the description of their official job duties and collaborate with their colleagues. A number of empirical studies have shown that employees who communicate with the organization's identity not only tend to go beyond their job duties but also tend to make more effort to promote the organization's collective interests and values (31-33). Finally, we hypothesize:

Hypothesis 2: Organizational identification has a direct relationship with organizational citizenship behavior.

### 1.4. The Mediating Role of Organizational Identification

Based on social identity theory, employees who identify strongly with their organization are more likely to engage in behaviors that benefit the organization (8). Therefore, it is expected that sustainable human resource management practices will lead to higher levels of OI, which in turn will lead to higher levels of organizational citizenship behavior.

As the organization's use of sustainable human resource management practices that benefit external stakeholders leads employees to identify more strongly with their organization, they begin to consider the organization's values and interests as their own and integrate them into their self-concept (17), which leads them to participate in extra-role activities, including organizational citizenship behavior, which in turn plays a pivotal role in the organization's goal attainment. These behaviors have a positive impact on the organization's image and provide employees with an opportunity to enhance their self-concept. Furthermore, as proposed by social exchange theory, mutual obligations emerge from the reciprocal interactions between two parties engaged in a relationship. Organizations initiate this social exchange by offering favorable treatment to employees along with economic or socio-emotional resources. Subsequently, employees experience a sense of obligation to respond in kind to the positive treatment they have received. This reciprocity takes the form of behaviors that directly contribute to the organization's well-being, such as engaging in OCB. In addition, social exchange theory suggests that obligations arise as a result of mutual social exchange between two parties in a relationship, and social exchange is initiated by organizations when they positively treat their employees and provide them with economic or socio-emotional resources. In turn, employees feel obligated to reciprocate the positive behavior bestowed upon them with behavior that directly benefits the organization, such as OCB (24).

2 Hence, organizations embrace s-HRM practices that directly contribute to employee welfare. These practices foster social exchange processes, which in turn prompt employees to cultivate heightened levels of organizational identity. Consequently, employees exhibit a psychological commitment to the organization as a response to this identity. (34). In the present study, we expect that higher levels of OI and the willingness of the organization to participate in a reciprocal social exchange process with its employees will lead to a reciprocation on their part by demonstrating organizational citizenship behavior.

Hypothesis 3: Organizational identification mediated the relationship between sustainable human resource management and organizational citizenship behavior.

**Figure 1.**
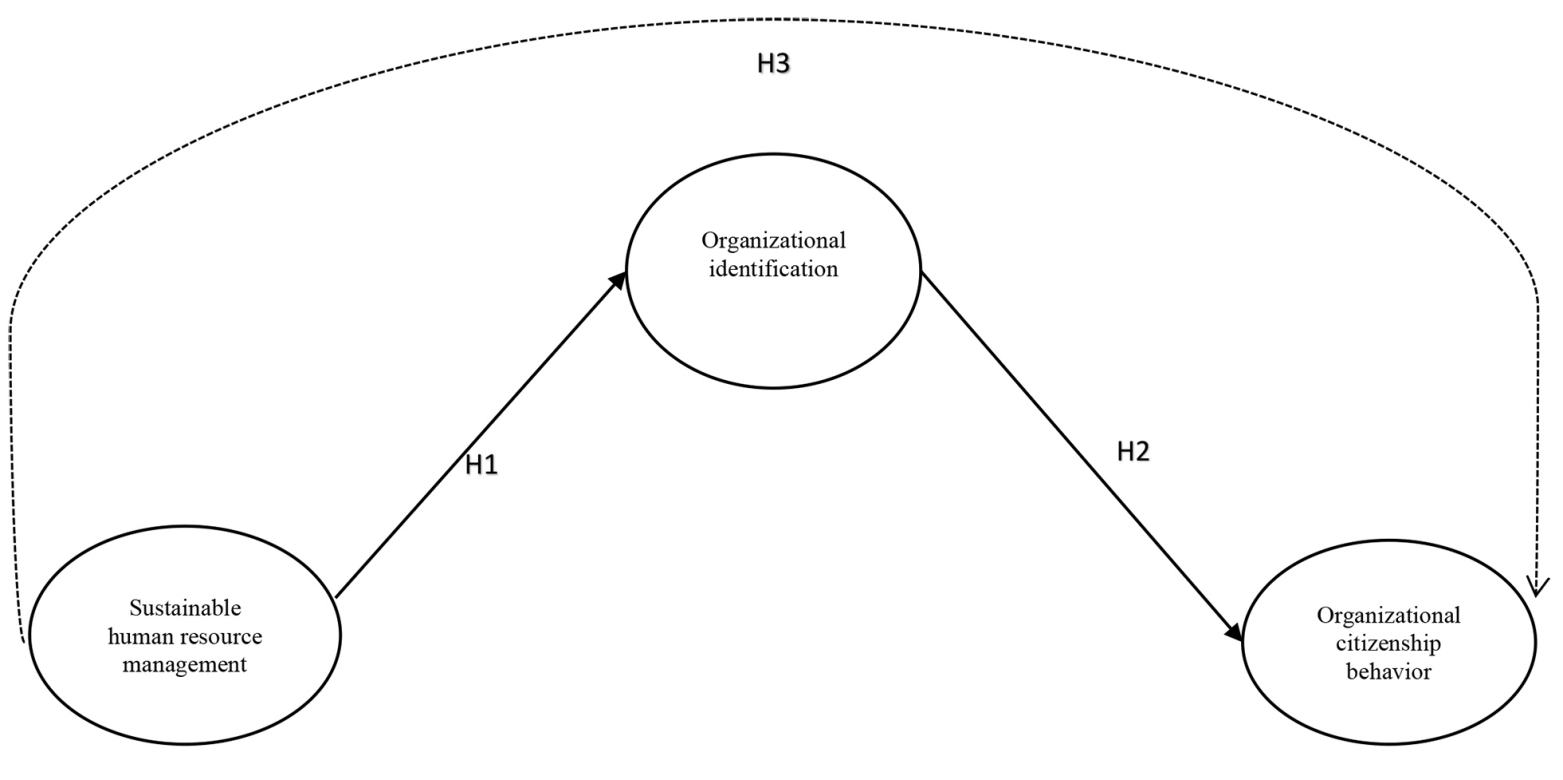
Conceptual Model

The conceptual model and all three hypotheses are displayed in Figure 1. 

## 2. Methods

### 2.1. Population and Sample

The study population comprised all employees in the research and development (R&D) departments of pharmaceutical companies in Iran. The sample was selected using the stratified random sampling technique to ensure that the selected sample was representative of the entire population. 

There were approximately 117 registered finished product manufacturing companies in the Iranian pharmaceutical sector. All of them were contacted and sent the questionnaires. Overall, 500 questionnaire surveys were distributed to all of their R&D departments, and finally, 316 completed questionnaires were collected. 

### 2.2. Measures

Data was collected using a structured, standard questionnaire, which was translated from English to Persian and then back-translated. The questionnaire comprised a combination of open-ended and close-ended questions. The close-ended questions were used to collect data on the study variables based on a five-point Likert scale (completely agree, agree, have no opinion, disagree, and completely disagree). In contrast, the open-ended questions were used to collect data on personal information, such as age, gender, education level, and job position (35).

#### 2.2.1. Sustainable Human Resource Management

A standard questionnaire based on De Prins et al. was used to measure sustainable human resource management. This questionnaire consists of 11 items that measure the organization's human resources management (36).

#### 2.2.2. Organizational Identification

To measure OI, a standard questionnaire based on the study of Mael and Ashforth (1992) was used (37). This questionnaire consists of 6 items (20 to 25 questions of the questionnaire) that measure OI based on a five-point Likert scale.

#### 2.2.3. Organizational Citizenship Behavior

A standard questionnaire based on the study of Podsakoff et al. was used to investigate the behavior of organizational citizens (29). This questionnaire consists of 20 items that measure OCB based on a five-point Likert scale.

### 2.3. Questionnaire Reliability and Validity

Cronbach's alpha coefficients were used to evaluate the reliability of the research instrument. Cronbach's alpha coefficients for all variables used in the current study were above 0.7, which is the acceptable level of preferred reliability (38) . Following the data analysis procedure using the partial least squares (PLS) algorithm, the factor loadings of the questionnaire items were measured. Subsequently, Cronbach's alpha coefficients were computed. Therefore, the questionnaire used in this research can be considered reliable. Convergent validity was used to confirm the validity of the measurement tool. For convergent validity, the average variance extracted (AVE) was calculated in order to confirm convergent validity.

The assessment of discriminant validity for the research constructs was carried out utilizing the Fornell-Lacker criterion (39). According to this prescribed approach, the square root of the average variance extracted (AVE) for each construct should exceed its correlation with other latent constructs. The constructs were significantly different from each other. Factor analysis was carried out to validate the data according to the criteria suggested by Kaiser (1958), i.e., an eigenvalue greater than 1 and an absolute loading factor value greater than 0.5 (40). All AVE scores were more than 0.5, reaching the acceptable level (41).

As shown in Table 1, Cronbach’s alpha coefficients for all variables used in the current study were above 0.7, which is the acceptable level of preferred reliability (38).

**Table 1.** Cronbach's Alpha Coefficients and AVEs to Demonstrate Reliability and Convergent Validity

	Cronbach's Alpha	AVE
**Whole questionnaire**	0.973	
**Sustainable human resource management dimension**	0.874	0.676
**Organizational identification dimension**	0.789	0.680
**Organizational citizenship behavior dimension**	0.922	0.659

The average variance extracted (AVE) scores for all three variables are also above the recommended threshold of 0.5 (Table 2), indicating that each construct captures a sufficient amount of variation in its respective set of items. Therefore, it can be concluded that the measures used in this study have adequate reliability and convergent validity.

**Table 2.** Discriminant Validity Using Fornell and Larcker’s Approach

	S-HRM	POS	OI	OJ	OCB
**S-HRM**	0.484				
**POS**	0.348	0.502			
**OI**	0.342	0.386	0.512		
**OJ**	0.406	0.339	0.320	0.480	
**OCB**	0.404	0.358	0.356	0.409	0.466

### 2.4. Data Analysis

The chosen approach for examining the theoretical assumptions using empirical data in this study was structural equation modeling (SEM). This method was favored due to its capability to comprehensively assess a theoretical model through the integration of both the measurement model and the structural model within a single analysis.

In this study, WarpPls 7.0 software was used for the fit of the model fitting and path analysis, which is based on the partial least squares (PLS) method. Warping is a non-linear data analysis technique used in partial least squares (PLS) path modeling. Warping allows for the detection of non-linear relationships between variables by transforming the data so that it becomes more linear. This means that the relationships between the latent variables are not constant across the range of the variables, and the strength of the relationship changes depending on the level of the variables (42).

## 3. Results

Taking in mind that the demographic characteristics of respondents might play a role in how they respond to questionnaires, we first report the demographic data. As displayed in Table 3, most of the respondents were female and had postgraduate education or higher. Also, about 73 percent of the respondents had work experience between zero and ten years, which is in line with the general characteristics of the Iranian pharmaceutical industry.

**Table 3.** Respondents’ Demographic Data

Variables	No. (%)
**Gender**	
Male	110 (34.8)
Female	206 (65.2)
**Age (y)**	
Less than 25	8 (2.5)
25 - 35	198 (62.7)
36 - 50	107 (33.9)
More than 50	3 (0.9)
**Education**	
Diploma and postgraduate diploma	3 (0.9)
Bachelor	55 (17.4)
Master	166 (52.5)
Pharm. D	57 (18.0)
PhD	35 (11.1)
**Job position**	
Junior specialist	5 (1.6)
Specialist	238 (75.3)
Senior specialist	44 (13.9)
Supervisor	26 (8.2)
Manager	3 (0.9)
**Job experience (y)**	
0 - 5	109 (34.5)
5 - 10	121 (38.3)
10 - 20	75 (23.7)
20 - 30	10 (3.2)
More than 30	1 (0.3)

To evaluate the presence of non-response bias, a comparison was made between the responses received in the early and late stages, following the suggestion by Armstrong and Overton (1977) (43). The outcome of the *t*-test demonstrated no significant disparity between these two response groups, thereby indicating the absence of non-response bias within this research.

To address potential common method bias, Harman's one-factor test was executed. The analysis revealed that only 24.70% of the overall variance could be accounted for by the single extracted factor, a proportion below 50%. These findings collectively signify that non-response bias was not evident in this study.

In order to check the fit of the model, APC, ARS, AARS, AVIF, GOF and SPR indexes were used.

The value of the APC index (average path coefficients) was equal to 0.257, and its significance level was equal to 0.001. Therefore, the model was evaluated as favorable from this perspective. The value of the ARS index was equal to 0.724, and its significance level was equal to 0.001. Therefore, from this point of view, the model was evaluated as desirable. Another fitness index used for the fitness of the model was the AARS index, whose value was equal to 0.720, and its significance level was equal to 0.001, so the model was evaluated as favorable from this point of view. Another fitness index used for the fitness of the model is the AVIF index, whose value is equal to 4.78, which is acceptable if it is less than 5. Therefore, from this point of view, the model was evaluated as desirable. Another fitness index used for the fitness of the model is the GOF index, whose value is equal to 0.851, which is acceptable and good if it is greater than 0.36. Another fitness index used for the fitness of the model is the SPR index (simpson's paradox index), whose value was equal to 0.875, which is acceptable and good if it is greater than 0.7. Therefore, from this point of view, the model was evaluated as favorable (Kock 2020).

The SEM results, as shown in Table 4, indicated that H1 and H2 were confirmed. To test the mediation effect (H3), first, the direct relationship of s-HRM and OCB was evaluated, and the results indicated that there was a direct relationship. Therefore, according to Kock (2011), H3 was supported (42). Lastly, to determine the degree of mediation, the variance accounted for (VAF) was calculated (44) at 0.249, which means that the mediation effect of OI on the s-HRM and OCB relationship was partial.

**Table 4.** Results of Hypotheses Testing

	Hypothesis	P-Value	ß	Result
**1**	Sustainable human resource management has a direct relationship with organizational identification.	< 0.001	0.493	Supported
**2**	Organizational identification has a direct relationship with organizational citizenship behavior.	< 0.001	0.364	Supported
**3**	Organizational identification mediated the relationship between Sustainable human resource management and organizational citizenship behavior.	< 0.001	0.18	Supported

## 4. Discussion

This research focused on examining the short-term outcomes of s-HRM and its relationship with OCB of employees in the R&D department of generic pharmaceutical companies in Iran. While the social exchange perspective addresses the impacts of sustainable human resource management on employee well-being and interests, the social identity viewpoint offers a more pragmatic interpretation of how s-HRM influences the establishment of social identity within Iranian pharmaceutical firms (14).

The context was chosen because OCB can play a critical role in pharmaceutical companies, especially in the R&D department, for several reasons: The R&D department is responsible for developing new products and technologies that drive innovation and growth in the industry, which takes a considerable amount of time to produce tangible outcomes (45). OCB can play a critical role in the success of the R&D department of pharmaceutical companies by promoting innovation, quality, efficiency, and employee well-being. Moreover, OCB can contribute to innovation by encouraging employees to share knowledge, take initiative, and collaborate with others, which can lead to new ideas and breakthroughs. In this regard, OCB can contribute to new product development by promoting behaviors such as attention to detail, adherence to procedures, and proactive problem-solving, which can reduce errors and improve the quality of work. Furthermore, it can contribute to efficiency by promoting behaviors such as time management, resource conservation, and willingness to go above and beyond expectations, which can improve productivity and reduce costs. Most importantly, the R&D process in the pharmaceutical industry can be demanding and stressful. OCB can contribute to employee well-being by promoting supportive and positive work environments (13).

This study found that s-HRM has a positive and significant direct relationship with OI, and OI has a positive and significant direct relationship with OCB and indicated that OI partially mediates the relationship between s-HRM and OCB. This means that when employees perceive that their organization values s-HRM practices, they are more likely to identify with their organization. S-HRM could be strengthened in organizations by providing training and development opportunities to help employees build skills and competencies that are aligned with the organization's goals and values and that can contribute to their sense of identity and commitment to the organization (46). In turn, this increased sense of OI leads to greater engagement in OCB. This increased sense of OI could be achieved through fostering a culture that promotes a strong organizational identity, creating opportunities for employee involvement and participation, and recognizing and rewarding employee contributions to the organization. The role of OI as a mediator between s-HRM and OCB is also consistent with previous research, as it has been found to mediate the relationship between various HR practices and outcomes, including OCB, job satisfaction, and organizational commitment (47). A practical way to make use of this finding in pharmaceutical companies is to incorporate s-HRM practices into organizations in order to improve employee well-being and job satisfaction, which may ultimately lead to better OI and higher levels of OCB.

A strong identification with the organization can help a person feel a sense of belonging to that organization. When employees have a strong organizational identification and tie their identity with the organizations, they see the organization's survival and success as their own. Therefore, a person has a greater desire to do activities that include more benefits of the organization instead of individual benefits, which are displayed as OCB (24). This suggests that strengthening employees' identification with their organization can be an effective way to promote positive behaviors and outcomes. This is in line with the body of research on the relationship between s-HRM and employee outcomes, including OCB. Additionally, if employees perceive more overlap between their identity and the organization's identity, they will identify more and more coherently with their organization. Hsieh et al. stated that identity can be defined in terms of the relationship that individuals have with their organization. The processes of forming and creating identity are completely relational or based on relationships. They are affected by interactions and signs that employees receive from others inside and outside the boundaries of the organization (48, 49). Creating interactions between employees and managers by facilitating vertical communication in the organization and broadcasting to employees that managers expect to receive their suggestions and ideas makes them think of the company's successes as their own. In other words, the formation of organizational identification is influential in providing learning opportunities for employees, paying attention to their work-related knowledge, and maintaining employability, making employees signal that the company considers them as more than a source to achieve organizational success and the company cares about them and their future as members of the organization (50).

The partial mediation effect of OI could be explained with a number of reasons. One possibility is that other unmeasured variables may also be mediating the relationship between s-HRM and OI. Another possibility is that the relationship between s-HRM and OI may depend on the specific context in which the research was conducted, such as the organizational culture and practices, the characteristics of the employees, or other external factors.

### 4.1. Conclusions

Based on the results of this study, it can be concluded that s-HRM practices have a positive impact on OI and, in turn, on OCB. Findings suggest that although social exchange theory and OI theory cannot completely explain how s-HRM that benefits internal stakeholders (such as employees) affects employees' willingness to go beyond their job role to engage in OCB, they provide a solid foundation for future research to more systematically investigate these issues in other sectors of the pharmaceutical industry and other industries. Despite the direct relationships that exist between mediation and positive outcomes, it is clear that mediation is partial in its effectiveness. This study has highlighted the limitations of mediation and the need for a more comprehensive approach to prompt OCB. As an academic essay, it is important to acknowledge the complexity of this topic and the need for further research and discussion. Ultimately, the insights gained from this essay can inform individuals and society as a whole in their pursuit of a more balanced work and life. 

### 4.2. Limitations

As with every other study, this study has a number of limitations that should be considered when interpreting the findings. The first limitation is that the study uses self-reported data, which may be subject to social desirability bias. Secondly, the study only focuses on R&D personnel in the pharmaceutical industry in Iran, and the findings may not be completely generalizable to other industries or countries with different cultural and organizational contexts. Lastly, the study is cross-sectional, and therefore, causality cannot be established between the study variables.

### 4.3. Ethical Considerations

This study complied with ethical considerations by ensuring that informed consent was obtained from all the participants before they were included in the study. Participants were informed about the purpose of the study and the data collection methods. The confidentiality and privacy of the participants were maintained by ensuring that the data collected was kept confidential and was not disclosed to any unauthorized persons.

## Data Availability

The dataset presented in the study is available on request from the corresponding author during submission or after publication.
